# Integrated bulk and scRNA sequence identified anoikis-related diagnostic biomarkers and potential association with immune infiltration in type A aortic dissection

**DOI:** 10.18632/aging.205126

**Published:** 2023-10-24

**Authors:** Kexiang Feng, Zhongwei Zhang, Jie Luo, Wenjie Wang, Tianjie Li, Jing Luo, Hongbo Huang

**Affiliations:** 1 Department of Cardiac Surgery, The First Affiliated Hospital of Kunming Medical University, Yunnan, China; 2 School of Clinical Medicine, Tsinghua University, Beijing, People’s Republic of China

**Keywords:** type A aortic dissection, anoikis-related genes, single-cell RNA sequence, diagnostic biomarker, immune-cell landscape

## Abstract

Type-A aortic dissection (TAAD) is common life-threatening cardiovascular diseases with high-morbidity and mortality but the concrete etiology of disease remains unclear, which might disturb or delay the early diagnosis for TAAD. Anoikis is a special form of programmed cell-death (PCD) induced by detachment of anchorage-dependent cells from the extracellular matrix (ECM) or neighboring cells, and has been widely applied to identify anoikis-related biomarkers for the prediction and prognosis in oncological fields. However, the specific roles of anoikis-related genes (ARGs) in TAAD remain unclear. In this study, we first identified and validated eight diagnostic ARGs for TAAD based on multiple RNA-sequence datasets, including *CHEK2, HIF1A, HK2, HMGA1, SERPINA1, PTPN1, SLC2A1* and *VEGFA.* The comprehensive functional annotation was evaluated by the integrated functional enrichments analysis. We identified the activation of inflammatory-related pathways, metabolic reprogramming and angiogenesis, and the inhibition of cardiovascular development pathways in TAAD. Immune cell infiltration (ICI) analysis further demonstrated that innate immune-cells were more dominant than adaptive immune-cells in TAAD tissues, especially in macrophages, monocytes, activated-DC, NKT cells and CD56+dim NK cells. The cellular landscape was further validated by single-cell RNA sequence technology with significant associations with anoikis in TAAD patients. Four vital ARGs (*HIF1A, HMGA1, SERPINA1* and *VEGFA*) were ultimately identified along with the changes of differentiation trajectory, and major expressions were conformably concentrated on Macro1-3, Mono1-2 and Mono4 subtypes. These findings provide a promising diagnostic biomarker for the accurately diagnosing the disease and would be helpful to further explore the potential pathogenesis with anoikis process for TAAD.

## INTRODUCTION

Aortic dissection (AD) is one of the most common life-threatening cardiovascular diseases characterized by a tear and bleeding of inner aortic layer and wall. The annual incidence of AD is approximately 3 to 6 cases per 100,000 people, and the incidence has been dramatically increasing in recent years [1]. Based on whether the ascending aorta is involved, AD is divided into type A aortic dissection (TAAD) and type B aortic dissection via the Stanford classification. Due to its abrupt onset and high mortality rate, the AD patients always exhibited a poor prognosis, especially for TAAD [2]. During the natural progression of TAAD, the mortality of untreated patients is approximately 32% in 24 hours, 50% in three days, 80% in next two weeks and up to 90% with aortic rupture [3]. However, the precise pathogenesis of TAAD remains unclear, and this disease condition is considered to be the result of a combination of multiple factors, including genetic, topographic anatomical, molecular biological, hemodynamic and immunological factors [4]. Therefore, there is an urgent need for understanding the potential etiology and screening novel biomarkers to improve the diagnosis and prognosis for TAAD.

AS a special form of programmed cell death (PCD), anoikis is characterized by the detachment of extracellular matrix (ECM) to avoid cells’ abnormal proliferating and growing after attaching to inappropriate matrix, which is essential for apoptosis [5]. Current studies have started to investigate the potential regulatory mechanism of anoikis through multiple intrinsic and extrinsic pathways, such as integrins, epidermal growth factor receptor (EGFR), TGF-β signaling, NF-κB signaling, hypoxia, reactive oxygen species (ROS), hippo pathway and so on [6, 7]. Matrix metalloproteinases (MMPs) have been reported to cause the formation of AD via promote degradation of elastin and collagen in ECM [8]. Through activating the HIF-1A pathway, hypoxia can promote the up-regulation of MMP-2/9 and further participate in the formation of AD [9]. In addition, several inflammatory factors, including TNFA and FASL, can also cause the caspase cascade in AD via binding to their corresponding receptors [10]. These studies indicated that anoikis might play an essential role in regulating the formation and progression of TAAD patients. There are increasing evidences that identifying anoikis-related genes (ARGs) as the novel biomarkers for the diagnosis of various diseases, such as tumor (lung adenocarcinoma, hepatocellular carcinoma) [11, 12], cardiovascular disease (ischemic stroke) [13] and so on. However, there is still lacking studies focusing on the role of ARGs in TAAD and exploring the potential correlations between ARGs and TAAD patients at cellular aspects.

In this study, based on the datasets of transcriptome sequencing, we firstly identified variant transcript patterns and further screened diagnostic ARGs for the diagnosis of TAAD patients, which were verified in multiple external datasets. In addition, potential targeted regulatory micro-RNAs were predicted to construct the complex regulatory networks for the diagnostic ARGs, and corresponding expression of miRNAs was successfully validated in datasets of non-code RNA-seq. Moreover, we comprehensively evaluated immune-cell landscapes and successfully validated them at cellular aspect via single cell RNA sequence (scRNA-seq) technology. Finally, we investigated the potential relationships of diagnostic ARGs and differentiation of vital cellular subtypes and further identified fatal ARGs in TAAD patients. In summary, our findings generated an in-depth understanding of ARGs diagnostic worth and the exploration of potential correlations between immune microenvironment and ARGs at single-cell levels, to help interpret possible anoikis-related mechanism for TAAD patients.

## MATERIALS AND METHODS

### TAAD datasets collection and preprocessing

A total of four transcriptome files were obtained from the Gene Expression Omnibus (GEO) database by searching the “type A aortic dissection” and “RNA sequencing” as the keywords (https://www.ncbi.nlm.nih.gov/geo/). These datasets included the sequencing data of aortic tissues samples from 27 TAAD and 24 HC cohorts, including GSE153434 (10 TAAD vs 10 HC), GSE98770 (6 TAAD vs 5 HC), GSE52093 (7 TAAD vs 5 HC) and GSE190635 (4 TAAD vs 4 HC) respectively. All these datasets were transformed into fragments per kilobase million/FPKM values and genes with mean expression<1 were eliminated. Subsequently, the probes were converted into corresponding symbols according to the platform annotation file and the data normalization was further conducted using the “normalizeBetweenArrays” function of Limma package [14]. The GSE153434 dataset was applied to perform subsequent analysis and the other three datasets were used as validation cohorts.

### Identification of variant transcript patterns for TAAD patients

To investigate the difference of transcript patterns between TAAD and healthy cohorts, we performed the multiple comparative analysis including principal component analysis (PCA), differential expressional genes (DEGs) identification and comprehensive functional enrichment analysis. The PCA was performed by the “factoextra” package (https://cloud.r-project.org/package=factoextra/) to expound the integral transcript difference for TAAD patients and the DEGs were defined with absolute log(fold-change/FC) >1 and adjusted *p*-value <0.05. The functional annotations of TAAD were conducted by Gene Ontology (GO) enrichment with ClusterProfiler package [15], Kyoto Encyclopedia of Genes and Genomes (KEGG) pathway enrichment with ClueGO plug-in in Cytoscape software [16] and Gene Set Enrichment Analysis (GSEA) with GseaVis package [17].

### Screening diagnostic anoikis-related biomarkers for TAAD patients

A total of 338 anoikis-related genes (ARGs) were obtained from the GeneCards database (https://www.genecards.org/), and ARGs with the relevance score >1 was comprised in our study. KEGG functional enrichment analysis was applied to assess the function of these differential ARGs. Differential expressional ARGs were further screened via intersecting DEGs with above ARGs. Subsequently, we performed univariate logistical [18], and LASSO regression [19] to select diagnostic ARG-related biomarkers for TAAD patients.

### Evaluating the diagnostic efficacy for ARGs and validation in external datasets

The pROC package was applied to evaluate the diagnostic efficacy of diagnostic markers with area under curve (AUC) values [20]. To further validate the diagnostic efficacy of markers, the corresponding expression of markers was compared between TAAD and HC cohorts in other external validation cohorts.

### Identification of targeted-regulatory miRNA for ARGs

To further screen potential targeted-regulatory micro-RNA (miRNA) of these diagnostic ARGs, we applied a powerful R package, called multiMiR, which included 8 predicted databases, 3 validated databases and 3 drug/disease-related databases [21]. Through setting parameter as “validated”, we identified diagnostic ARGs-related regulatory miRNAs with experiment’s validation. The regulatory networks between miRNAs and ARGs were constructed by the Cytoscape software [22] and the expressional levels of these miRNAs in TAAD patients were further validated in GSE190635 with non-code RNA sequencing.

### Immune cell infiltration analysis for TAAD

Subsequently, we systematically evaluated the comprehensive characteristics of immune microenvironment in TAAD tissues via a single-sample gene set enrichment analysis (ssGSEA) algorithm of GSVA package based on 28 immune cells [23]. The enrichment scores of each cell were then compared between TAAD and HC cohorts using boxplots generated by the ggplot2 package [24].

### Quality control and scRNA-Seq data pre-processing

Moreover, to validate the immune microenvironment and further explore the potential correlation between immune cells and ARGs, we gained a current single-cell RNA sequencing (scRNA-seq) dataset (GSE213740) to perform related analysis and validation, including 6 TAAD patients and 3HC cohorts. We downloaded the normalized 10X files with expressional matrix, cell barcodes and features in each sample, and then created the seurat object by the Read10X and CreateSeuratObject function of Seurat package [25]. Low-quality cells with over 5,000, below 200 expressed genes or more than 10% of mitochondrial and hemoglobin-related genes were removed. DoubletFinder package [26] was applied to remove double cells with an expected doublet rate of 0.075 and Harmony package [27] was used to remove the batch effects among individuals with RunHarmony function.

### Cellular subtypes identification and functional annotation

To identify cellular clusters, we selected 2000 highly-variable genes to perform the PCA dimensionality reduction and applied the RunUMAP function to conduct Uniform Manifold Approximation and Projection (UMAP) dimension reduction with 30 principal components. Then, the cellular clusters were identified by FindClusters function with resolution as 0.3. Cell clusters were further annotated based on the comparison between cluster’s markers and Zhang et al. annotated markers. The expression of cellular markers was exhibited in bubble diagram by DotPlot function and cellular proportions among groups were further compared to validate the results of ssGSEA. Based on the Wilcoxon rank sum test, DEGs of each cell were also identified between TAAD and HC groups using the “find_diff_genes” function of Scillus package (https://github.com/xmc811/Scillus) and functional annotation of each cell in TAAD patients was performed by GSEA using the test_GSEA and plot_GSEA function.

### Differentiation trajectory analysis with comparison of anoikis

Anoikis scores were calculated for each cell using the AddModuleScore function based on above diagnostic ARGs, and scores were further compared in each cell to identify anoikis-related cells in TAAD. To further expound the intercellular differentiation trajectory with changes of anoikis scores, we applied cell trajectory reconstruction analysis using the monocle2 package [28].

### Statistical analysis

All related statistical analysis was performed in R software (version 4.1.6). The continuous variables were exhibited as mean ± standard deviation and the comparison between groups was tested with Wilcox test. The statistical significance was considered with two-tailed adjusted *p*-value <0.05.

### Availability of data

Publicly available datasets were analyzed in this study. This data can be found here: Gene Expression Omnibus (GEO) (https://www.ncbi.nlm.nih.gov/geo/) (Accessions: GSE153434, GSE98770, GSE52093 and GSE190635); The scRNA-seq dataset was obtained from the GSE213740 datasets.

## RESULTS

### Abnormal transcript patterns in TAAD patients

**Figure 1:**
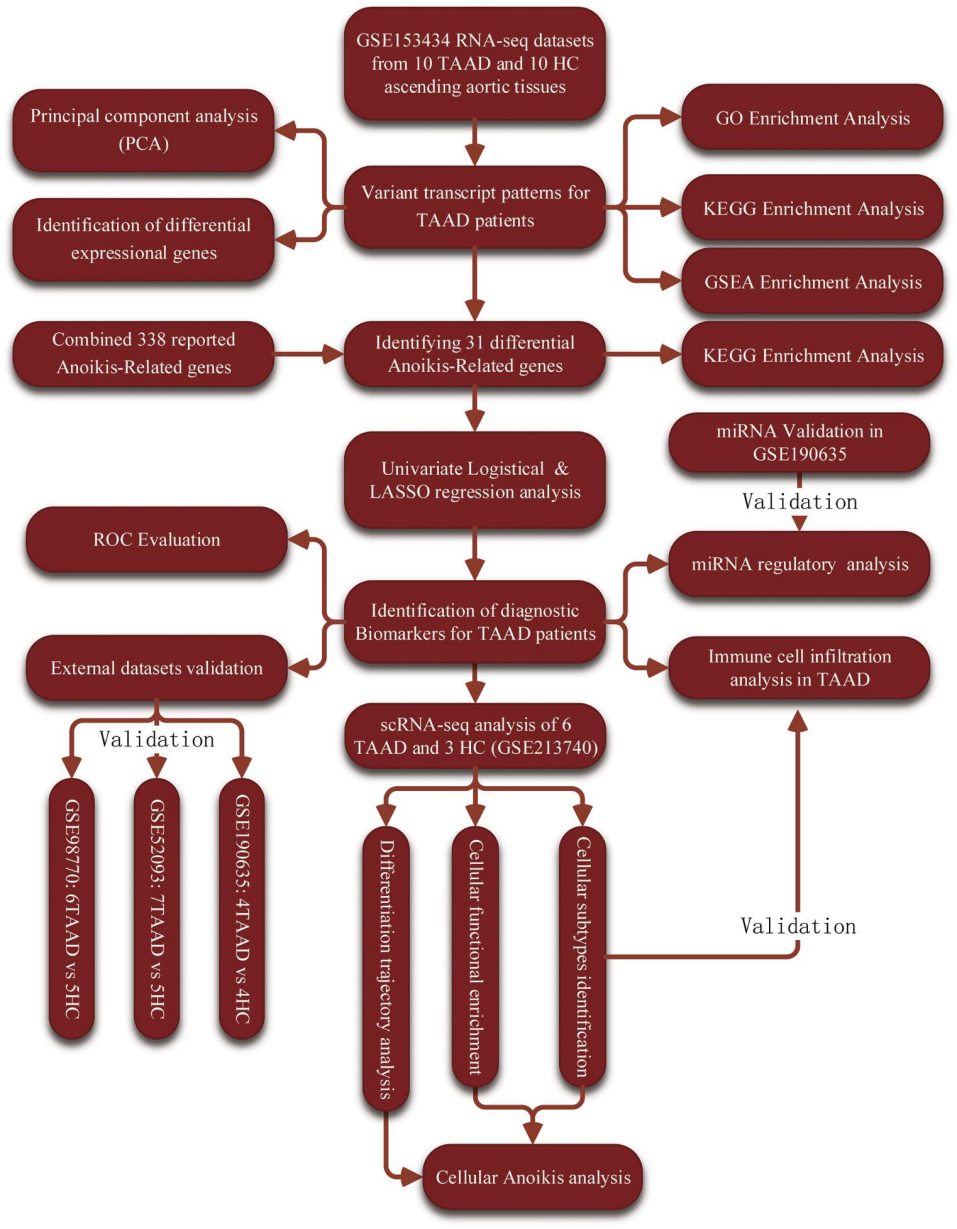
The workflow chart of this study.

**Figure 2:**
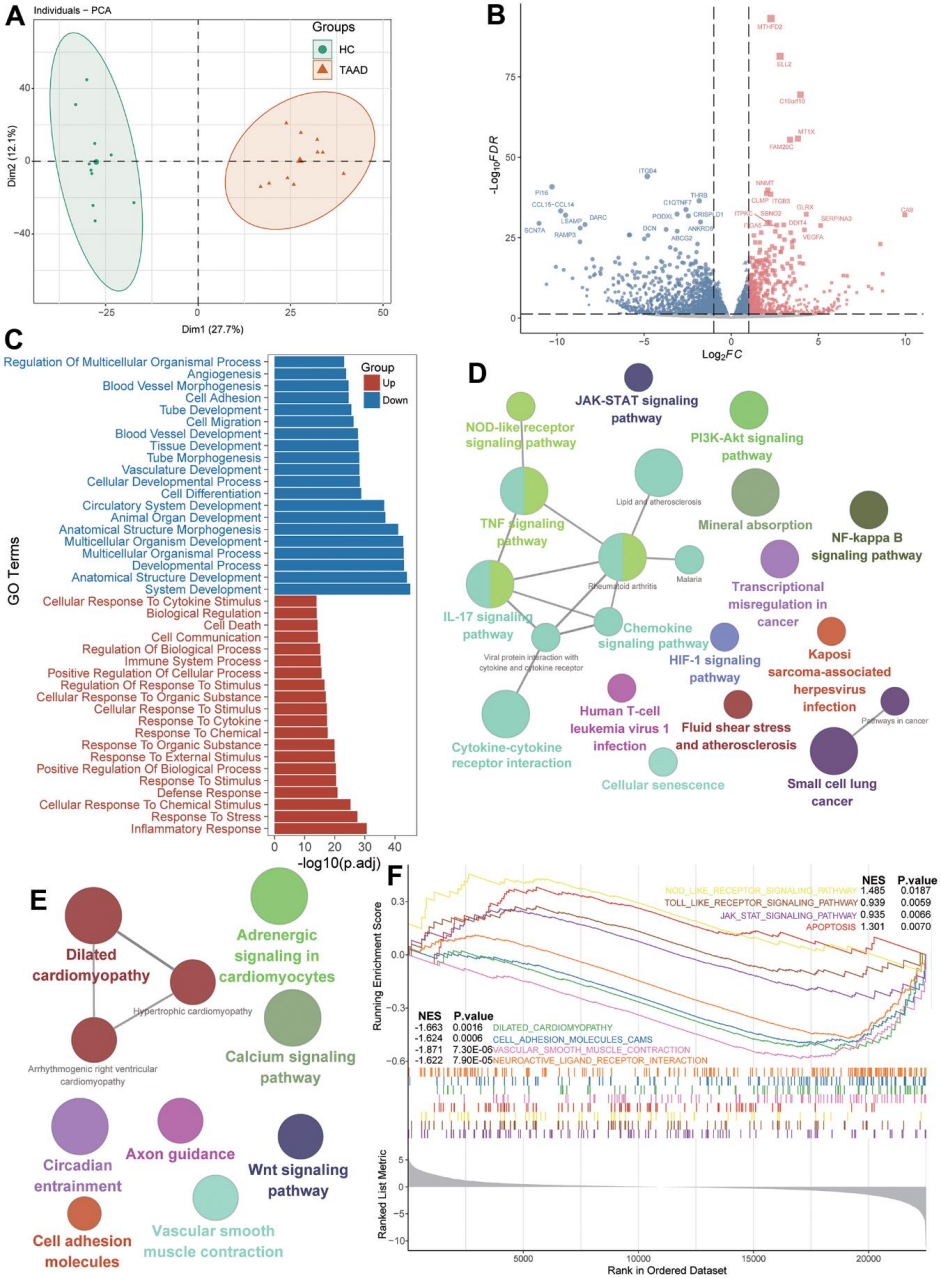
**Identification of DEGs and functional annotation for TAAD patients.** (**A**) Principal component analysis showing transcriptome signatures sorted TAAD and control cohorts into two clusters. (**B**) Volcano plots showing the DEGs between TAAD and HC cohorts with *p*<0.05 and absolute log (FC) >1. (**C**) The GO functional analysis of upregulated and downregulated DEGs. (**D**, **E**) The KEGG functional analysis of up-regulated and downregulated signatures. (**F**) The GSEA indicating the functional difference between TAAD and HC cohorts.

The workflow diagram of this study was displayed in the Figure 1 and datasets used in this study were summarized in Supplementary Table 1. To explore the integral transcript abnormities of TAAD patients, we conducted the PCA analysis and it revealed that TAAD and HC cohorts were significantly distributed in two different cohorts (Figure 2A). A total of 578 up-regulated and 1058 down-regulated DEGs were identified for TAAD and exhibited in the volcano plots with top genes (Figure 2B and Supplementary Tables 2, 3). To further explain the functional annotation of DEGs for TAAD, we performed the comprehensive analysis including GO, KEGG and GSEA enrichments. The GO enrichment analysis exhibited various response-related biological processes were significant activated in TAAD, such as Inflammatory Response, Response to Stress, Response to Cytokine, Response to Stimulus and so on. Contrastively, structure development-related processes were obviously inhibited in TAAD patients, including System Development, Anatomical Structure Development and Developmental Process (Figure 2C and Supplementary Table 4). In addition, we further demonstrated inflammatory-related pathways were significant enriched including TNF, JAK-STAT, IL-17 signaling, Cytokine-cytokine receptor interaction and NF-kappa B signaling pathway, while cardiomyocyte-related pathways were down-regulated, including Dilated cardiomyopathy, Adrenergic signaling in cardiomyocytes, Calcium and Wnt signaling pathway (Figure 2D, 2E and Supplementary Table 5). Using all genes with logFC in each pathway, the GSEA further validated the activation of JAK-STAT signaling, NOD-like and Toll-like receptor signaling, and Apoptosis in TAAD patients, with a coinstantaneous inhabitation of Dilated cardiomyopathy, Cell adhesion molecules, Vascular smooth muscle contraction (Figure 2F and Supplementary Table 6). All these pathways were enriched with high NES scores and significant adjusted *p*-values.

### Identification of diagnostic ARGs for TAAD patients

**Figure 3:**
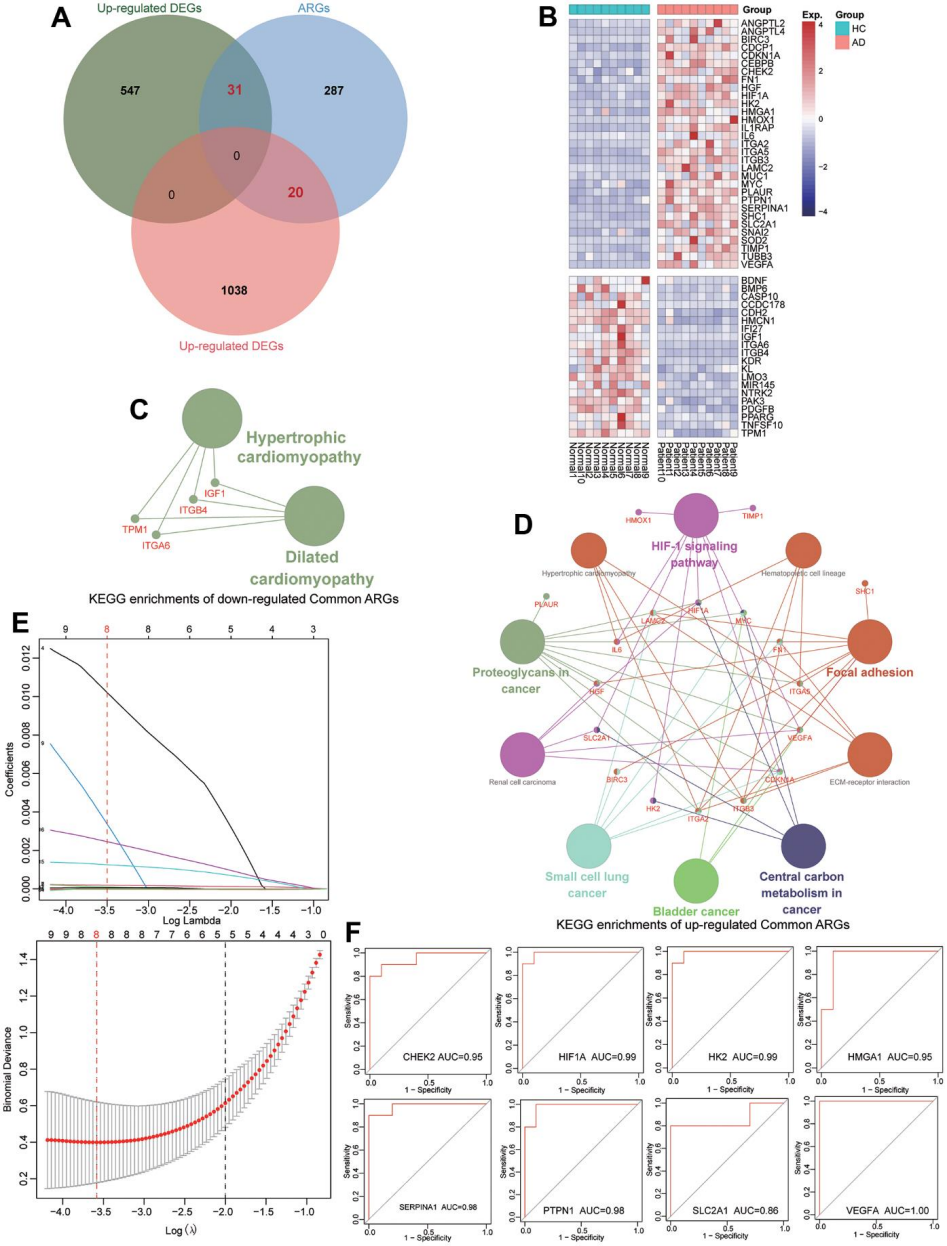
**Identification of diagnostic ARGs and ROC evaluation for TAAD patients.** (**A**) Veen plots identified 31 up-regulated and 20 down-regulated differential ARGs. (**B**) The expression of differential ARGs between TAAD and HC tissues in heatmaps. (**C**, **D**) The KEGG functional analysis of up-regulated and downregulated differential ARGs. (**E**) The results of LASSO regression screening the eight diagnostic ARGs for TAAD. (**F**) The ROC analysis revealed that these diagnostic ARGs exhibited prominent diagnostic efficiency for TAAD patients with high AUC values including CHEK2/0.95, HIF1A/0.99, HK2/0.99, HMGA1/0.95, SERPINA1/0.98, PTPN1/0.98, SLC2A1/0.86 and VEGFA/1.0.

To further identify the diagnostic anoikis-related markers for TAAD patients, a total of 338 known ARGs were used to be intersected with DEGs (Supplementary Table 7). Subsequently, 31 up-regulated and 20 down-regulated ARGs were selected and their expressional levels exhibited significant divisional capacity for TAAD patients (Figure 3A, 3B). The KEGG enrichment analysis revealed that these up-regulated ARGs were correlated to HIF-1 signaling pathway, ECM-receptor interaction, Focal adhesion and cancer-related pathways, but the down-regulated ARGs were enriched in Dilated cardiomyopathy and Hypertrophic cardiomyopathy (Figure 3C, 3D). After filtering with univariate logistical regression and LASSO regression, 8 diagnostic ARGs were ultimately ensured for TAAD including CHEK2, HIF1A, HK2, HMGA1, SERPINA1, PTPN1, SLC2A1 and VEGFA (Figure 3E).

### ROC evaluation and validation of diagnostic ARGs

**Figure 4:**
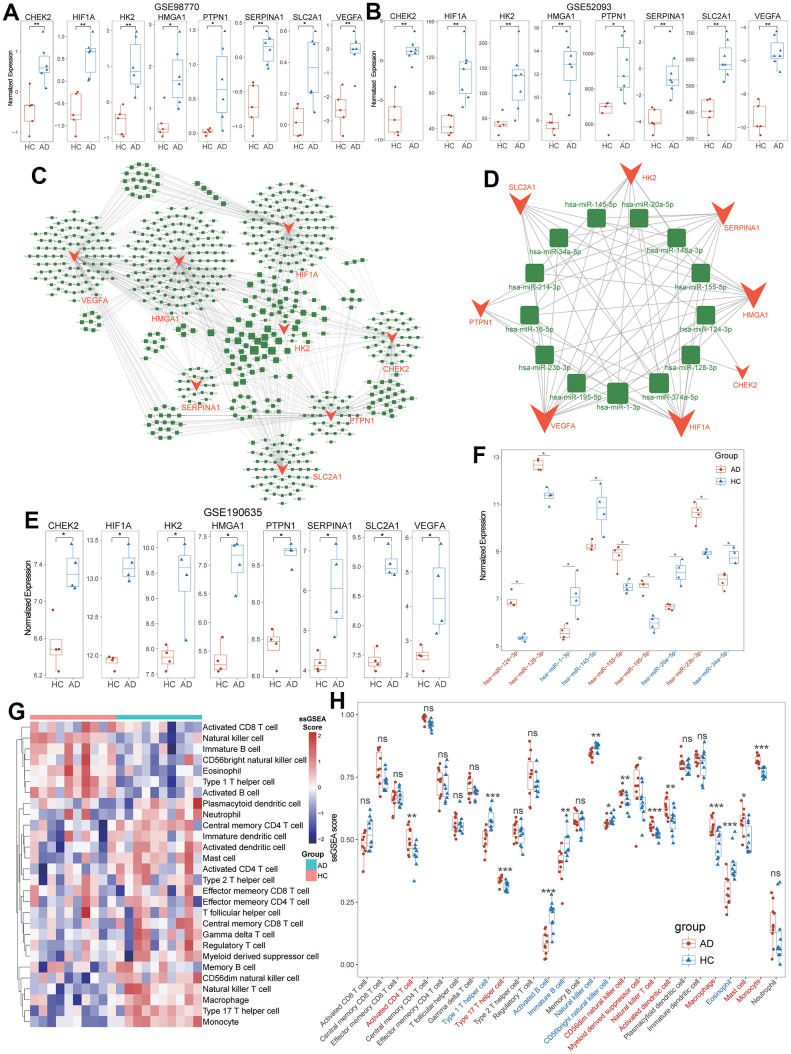
**Definition and validation of targeted miRNA for diagnostic ARGs.** (**A**, **B**) Validation of higher expression of diagnostic ARGs in GSE98770 and GSE52093. (**C**) Global between diagnostic ARGs and predicted miRNAs. (**D**) The vital regulatory network of common miRNAs to regulate the diagnostic ARGs. (**E**) Validation of higher expression of diagnostic ARGs in non-code RNA dataset (GSE190635). (**F**) Validation of the expressional levels of common miRNAs in TAAD based on GSE190635. (**G**) The heatmap of 28 immune cells’ ssGSEA scores between TAAD and HC cohorts. (**H**) The boxplot demonstrated the activation of macrophages, monocytes, activated dendritic cells and CD56+ dim NK cells, and the inhabitation of Th1 cells, B cells and CD56+ bright NK cells.

The ROC analysis revealed that these diagnostic ARGs exhibited prominent diagnostic efficiency for TAAD patients with high AUC values including CHEK2/0.95, HIF1A/0.99, HK2/0.99, HMGA1/0.95, SERPINA1/0.98, PTPN1/0.98, SLC2A1/0.86 and VEGFA/1.0, respectively (Figure 3F). To further validate the expressional difference of these diagnostic ARGs, we applied other 3 external datasets for TAAD. Due to the limitation of sample numbers, we didn’t perform the ROC analysis. All these markers exhibited higher expression in TAAD tissues than that of HC cohorts, including GSE98770, GSE52093 and GSE190635 (Figure 4A, 4B, 4E).

### Definition and validation of Targeted miRNA for diagnostic ARGs

Moreover, we successfully defined 651 potential miRNAs to regulate the expression of 8 diagnostic ARGs and constructed a regulatory network via multiMiR package (Figure 4C). Through the intersected miRNAs with diagnostic ARGs, a total of 13 targeted miRNAs were screened in an elaborate network (Figure 4D and Supplementary Table 8). We then applied the GSE190635, a dataset including non-code RNA sequence, to validate the differential expression of targeted miRNAs. It revealed 5 miRNAs (has-miR-124-3p, has-miR-128-3p, has-miR-155-5p, has-miR-195-5p and has-miR-23b-3p) were up-regulated and 4 miRNAs (has-miR-1-3p, has-miR-145-5p, has-miR-20a-5p and has-miR-34a-5p) were down-regulated in TAAD patients compared to HC cohorts (Figure 4F). The remaining 4 miRNAs were not detected in this dataset and these results implied the complex potential regulatory relationship between miRNAs and diagnostic ARGs in TAAD patients.

### Immune cell landscapes in TAAD

The infiltration of 28 immune cells were summarized in Figure 4G, 4H. Compared to HC cohorts, the TAAD patients exhibited higher infiltration scores in innate immune-associated cells, including macrophages, monocytes, activated dendritic cells and CD56+ dim NK cells. For the adaptive immune-associated cells, the TAAD patients exhibited mild infiltrate difference with activation of activated CD4+ T cells and type 17 T helper (Th17) cells, and the inhabitation of Th1 cells, activated B cells and immature B cells (Figure 4G, 4H and Supplementary Table 9). These findings indicated the infiltration of immune cells in TAAD was mainly characterized by innate immune-associated cells, especially for macrophages and monocytes.

### Cellular landscapes validation and anoikis scores estimation

**Figure 5:**
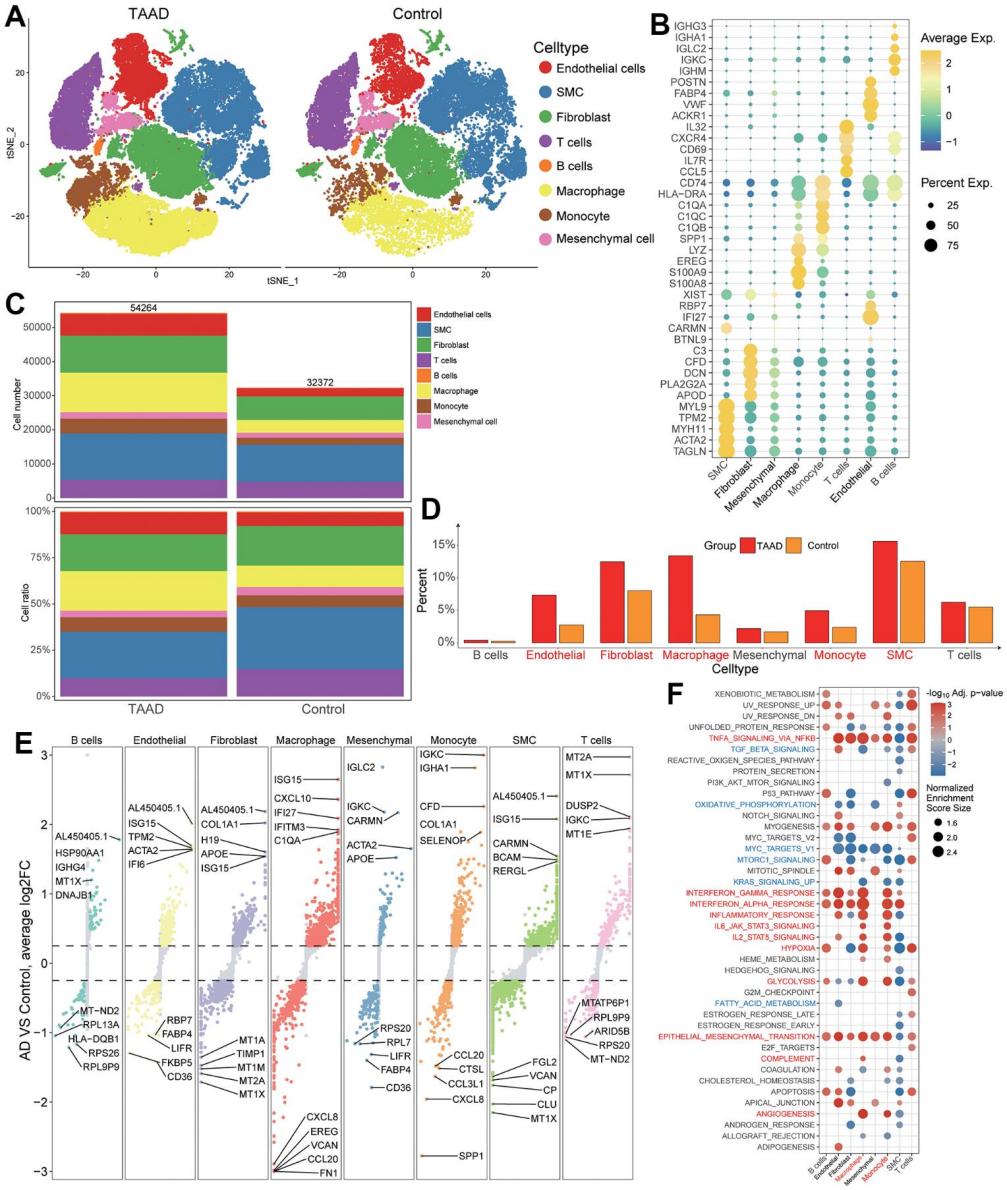
**Validation of immune cell landscapes through scRNA-seq.** (**A**) Integral cellular distribution between TAAD and HC cohorts; (**B**) The top5 corresponding cellular markers of each cell; (**C**, **D**) The comparison of cellular numbers and ratio between TAAD and HC in each cell; (**E**) The DEGs of TAAD in each cell. (**F**) the GSEA of each cell between TAAD and HC cohorts.

By evaluating cell type specific gene expression, we identified a total of 8 cell types in aortic tissues including 3 stroma cells (endothelial cells, smooth muscle cells (SMC) and fibroblasts) and 5 immune cells (T cells, B cells, macrophage, monocyte and mesenchymal cells) (Figure 5A). The top 5 cellular markers were displayed in bubble diagram and exhibited their specificity for each cell (Figure 5B and Supplementary Table 10). Through counting the cellular numbers and ratio, we found the difference between TAAD and HC cohorts was focused on the increase of endothelial cells, fibroblasts, macrophages, monocytes and SMC while the difference of B cells, Mesenchymal cells and T cells was not obvious, consistent with the cellular infiltration of ssGSEA analysis (Figure 5C, 5D).

The DEGs of each cell were further identified, and we found interferon-related genes (ISG15, IFI27, IFITM3) and immunoglobulin-related genes (IGKC, IGHA1) were upregulated in macrophages and monocytes of TAAD patients respectively (Figure 5E). The GSEA also demonstrated the activation of inflammatory and metabolic pathways in endothelial cells, fibroblasts, B cells, macrophages and monocytes of TAAD, including interferon-gamma/alpha response, TNFA signaling via NFKB, inflammatory response, IL6-JAK-STAT3 signaling, Hypoxia and Glycolysis. In addition, epithelial-mesenchymal transition and angiogenesis were also enriched but several signal transduction pathways were down-regulated in TAAD, such as TGF-Beta signaling, MTORC1 signaling, oxidative phosphorylation and so on (Figure 5F).

**Figure 6:**
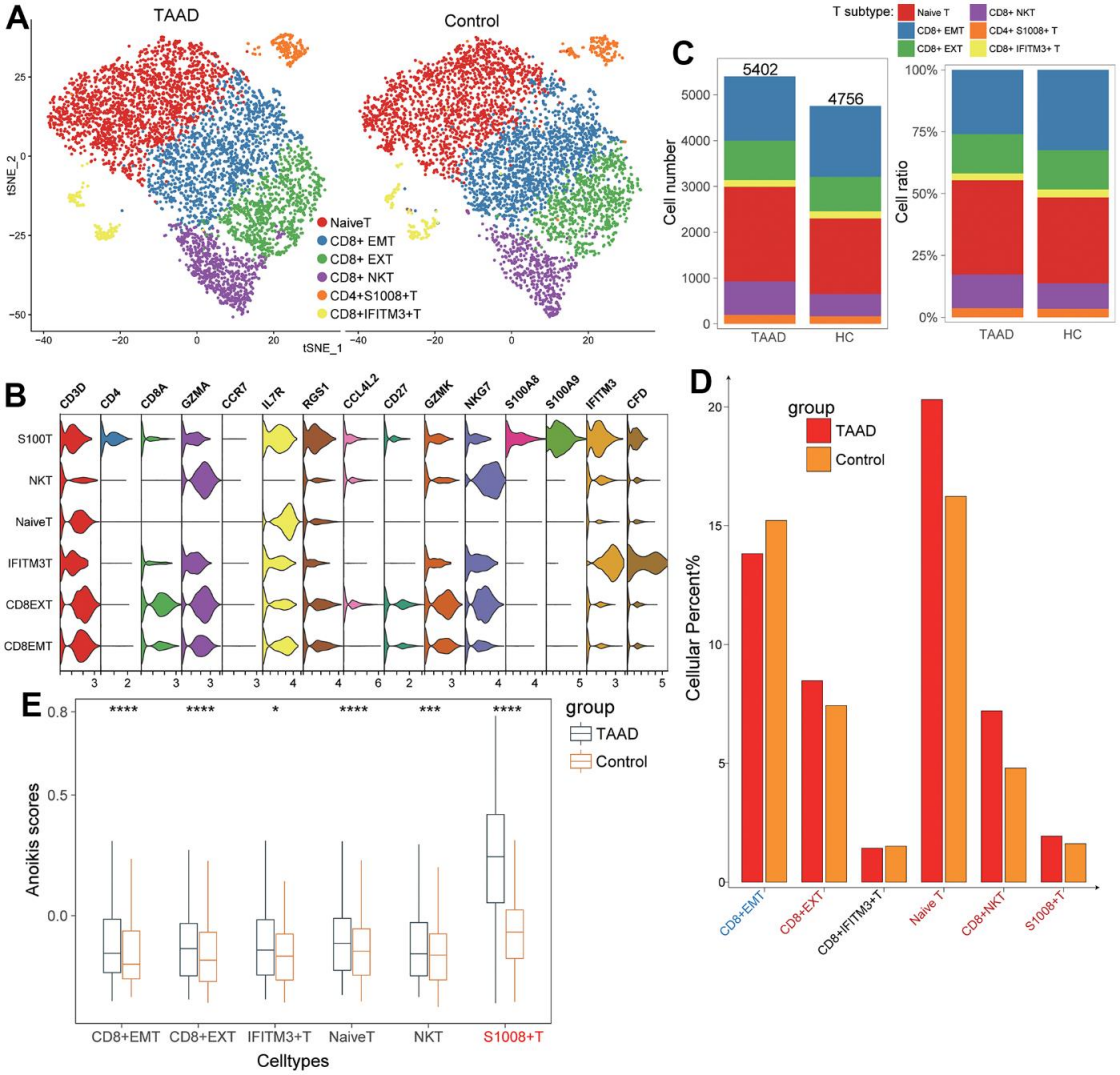
**Validation of T cells subtypes for ssGSEA algorithm.** (**A**) Identification of six T-cell subtypes between TAAD and HC; (**B**) The corresponding cellular markers with high-expression in each cell; (**C**, **D**) The comparison of cellular numbers and ratio between TAAD and HC in each cell; (**E**) The comparison of anoikis scores between TAAD and HC in different T cell subtypes.

To validate the landscape characteristics of T cells in TAAD, we further divided T cells into various cellular subtypes and compared the cellular ratio between TAAD and HC groups. After subjected to dimension reduction, T cell profiles were divided into 6 clusters and annotated as naïve T, CD8+ effector memory T (EMT), CD8+ exhausted T (EXT), CD8+ NKT, CD4+ S100A8+ T and CD8+ IFITM3+ T cells (Figure 6A). The violin plot showed that cellular markers were coincident with subtypes, such as IL7R and CCR7 for naïve T cells, GZMK and GZMA for NKT and CD8+ EXT cells and so on (Figure 6B). CD8+ EMT cells were found significantly decreased while other 4 cells were prominently increased in TAAD patients compared to HC cohorts (Figure 6C, 6D). All these T cells exhibited higher anoikis scores in TAAD patients, especially in S100A8+ T cells, suggesting the potential correlation between T cells and anoikis (Figure 6E).

### Potential relationships between macrophages and anoikis scores in TAAD

**Figure 7:**
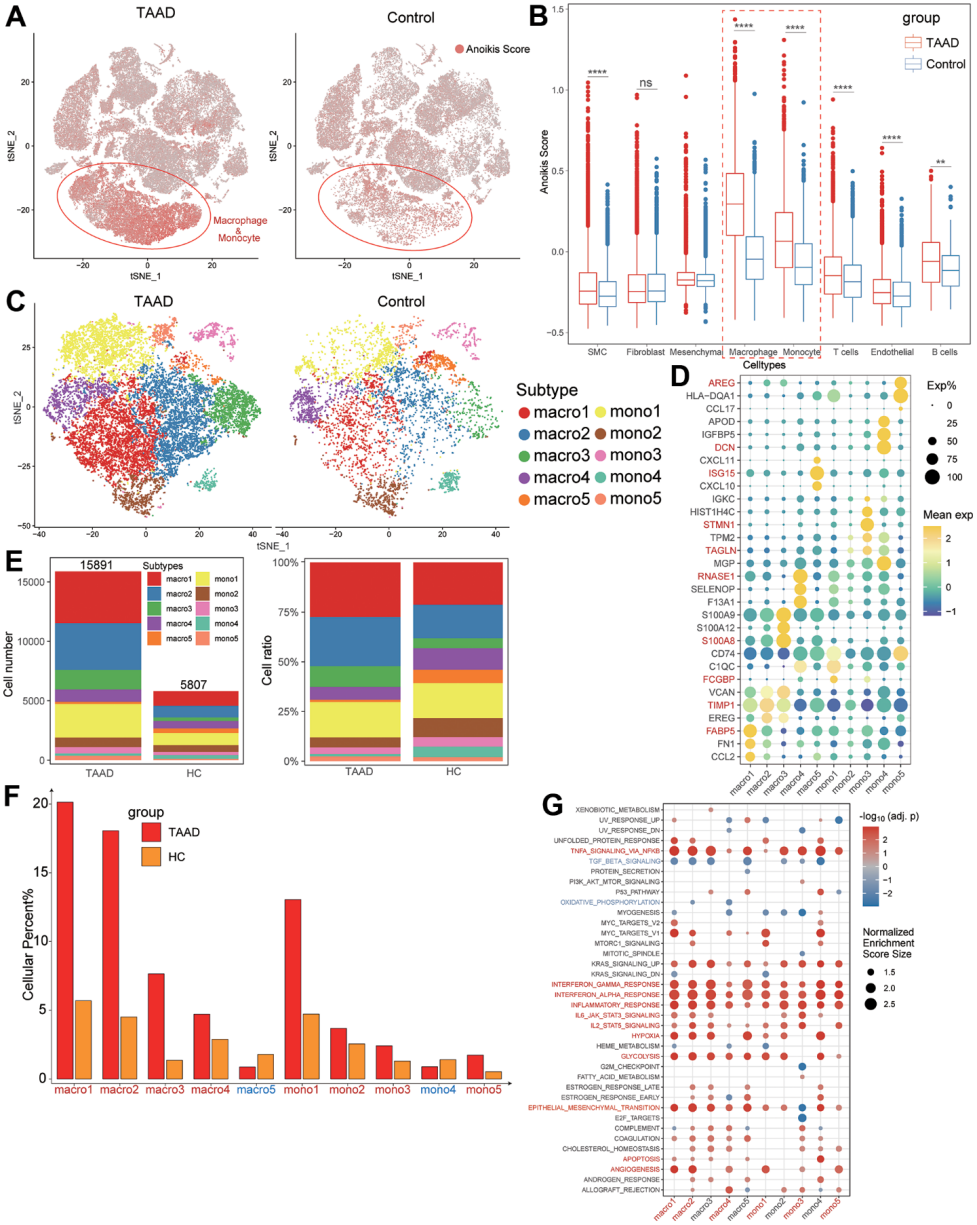
**Anoikis scores estimation a vital macrophage subtypes identification.** (**A**, **B**) The comparison of anoikis scores between TAAD and HC in all cell subtypes showing macrophages and monocytes were major cells for high anoikis scores; (**C**) Identification of five macrophage subtypes and five monocyte subtypes between TAAD and HC; (**D**) The corresponding cellular markers with high-expression in each subtype; (**E**) The comparison of cellular numbers and ratio between TAAD and HC in each subtype; (**F**) The GSEA of each macrophage and monocyte subtype between TAAD and HC cohorts.

We also compared the levels of anoikis scores between TAAD and HC groups in each cellular subtype and indicated the elevation of scores was primarily focused on macrophages and monocytes (Figure 7A, 7B). Moreover, we further defined 10 classifications of macrophages and monocytes (5 subtypes for each cell) and annotated them with significant markers, including FCGBP+ mono1, TAGLN+ mono2, STMN1+ mono3, DCN+ mono4, AREG+ mono5, FABP5+ macro1, TIMP1+ macro2, S100A8+ macro3, RNASE1+ macro4 and ISG15+ macro5 (Figure 7C, 7D). Except macro5 and mono4, the cellular ratios of most macrophages and monocytes were significant elevated in TAAD patients, particularly in macro1-4 and mono1 (Figure 7E, 7F). The GSEA further demonstrated that macrophages and monocytes were major vital cells for TAAD, with the coincident activation of multiple inflammatory and metabolic signaling pathways (Figure 7G).

### Differentiation trajectory analysis of macrophages and diagnostic ARGs

**Figure 8:**
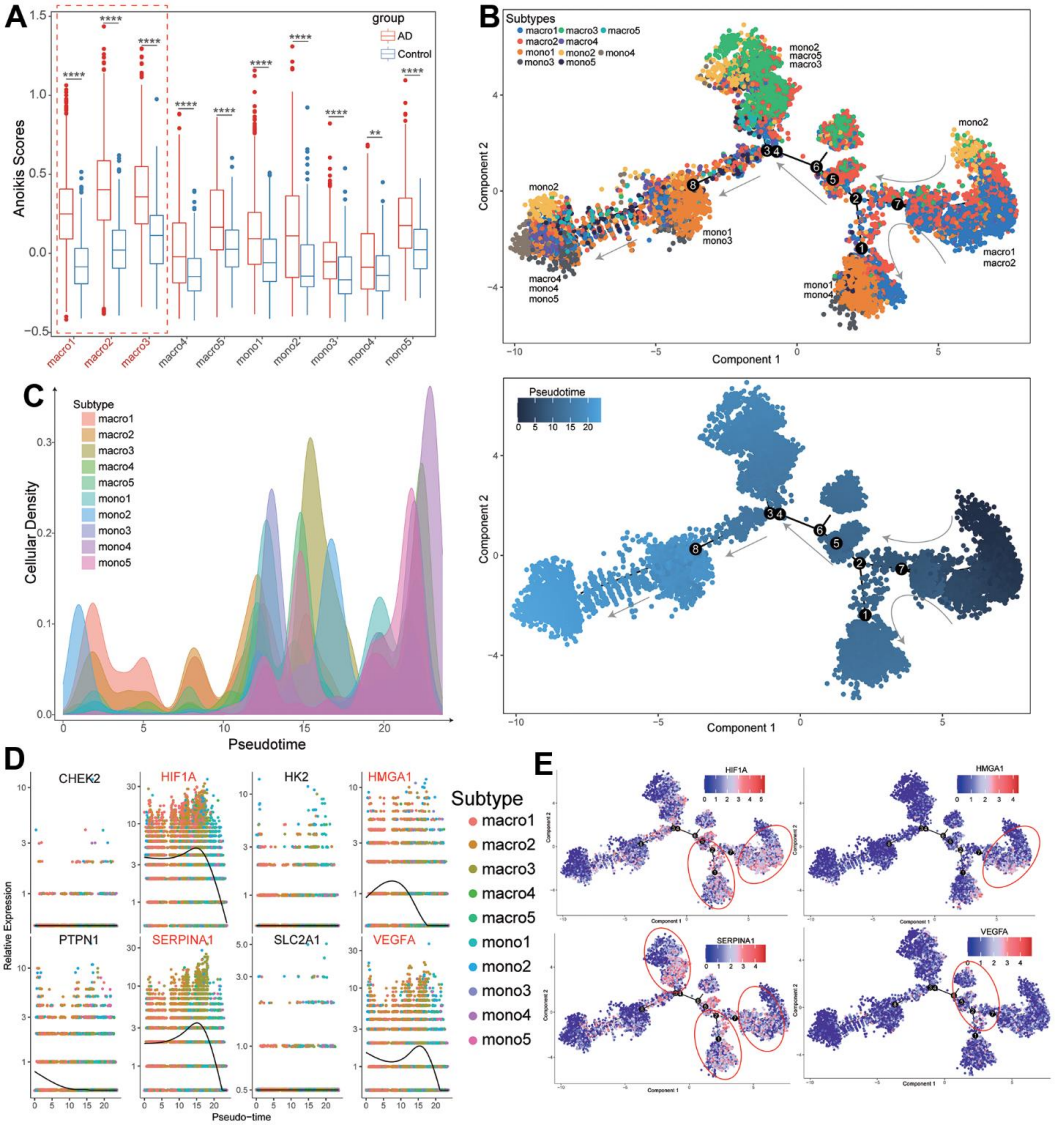
**Differentiation trajectory analysis of macrophages subtypes with expression of diagnostic ARGs.** (**A**) The comparison of anoikis scores between TAAD and HC in different macrophage and monocyte subtypes. (**B**, **C**) The differentiation trajectory of macrophage and monocyte subtypes with pseudotime. (**D**) The expression of eight diagnostic ARGs in macrophage and monocyte subtypes with pseudotime. (**E**) The trajectory distribution of four vital diagnostic ARGs in macrophage and monocyte subtypes of TAAD patients.

Notably, most macrophage and monocyte subtypes of TAAD patients exhibited higher levels of anoikis scores than HC cohorts, especially for macro1-3 subtypes (Figure 8A and Supplementary Table 11). The pseudotime trajectory analysis further revealed the arrangement of these cellular subtypes formed a certain differentiation rule based on its spatial relationships. Concretely, the trajectory analysis revealed that macro1 and macro2 were distributed at the origination of the trajectory and the differentiated into mixed cells of cluster1 (mono1 and mono4) and cluster2 (mono2, macro3 and macro5). The cluster3 (mono1 and mono3) were distributed at the middle while the cluster4 (macro4, mono4 and mono5) were distributed at end of the trajectory (Figure 8B, 8C). Finally, we further evaluated the pseudotime expressional changes of 8 diagnostic ARGs along with different macrophage and monocyte subtypes in TAAD patients. It revealed that 4 vital ARGs were high-expressed in the start and middle of the differentiation trajectory and significantly decreased at the end of trajectory, including HIF1A, HMGA1, SERPINA1 and VEGFA (Figure 8D). The trajectory plots further demonstrated the corresponding differential trajectory between cellular subtypes and vital ARGs and major expressions were concentrated on macro1-3, mono1-2 and mono4 subtypes, implying these cells might participate in the activation of ARGs in TAAD patients (Figure 8E).

## DISCUSSION

As a life-threatening abrupt cardiovascular emergency, TAAD patients always exhibit poor prognosis with high mortality. However, the precise pathogenesis of TAAD remains still unclear, leading to delaying the early diagnosis and intervention of disease, thus identification of novel reliable objective indicators for accurate diagnosis for TAAD is urgently needed. Anoikis is a special form related to apoptosis, characterized by anchorage cells’ detachment from ECM or adjacent cells, resulting in the incompatibility with the tissue’s microenvironment. Recently, studies have indicated the potential regulatory mechanism of anoikis on TAAD through multiple pathways, including ECM’s degradation, hypoxia, oxidative stress and proinflammatory factors. Therefore, we infer anoikis-related genes might be suitable as candidates for the precise diagnosis for TAAD and associated with the abnormal immune microenvironment of TAAD.

Through variant transcript analysis, we identified massive DEGs for TAAD patients and these DEGs were significant enriched in the activation of inflammatory signaling and response to stimulus or stress, including TNF, JAK-STAT, IL-17 signalings, Cytokine-cytokine receptor interaction, NF-kappa B signaling pathway, and Fluid shear stress and atherosclerosis. In contrast, cardiomyocyte-related pathways were significantly inhibited in these patients, including Dilated cardiomyopathy, Adrenergic signaling in cardiomyocytes, Vascular smooth muscle contraction, Calcium and Wnt signaling pathway. Wu et al. demonstrated that degradation of ECM was associated with the development of AD and might serve as a potential target for the treatment of AD [29]. Moreover, VSMCs participate in the pathogenesis of AD via degrading ECM and weakening the aortic wall, along with increasing MMP production and pro-inflammatory responses [30]. Our results of function enrichment were consistent with above studies and well interpreted complex pathogenesis of AD.

To further screen diagnostic ARGs for TAAD, we combined DEGs with ARGs and performed the machine learning of logistical and LASSO regression based on the 51 different expressional ARGs. A total of 8 diagnostic ARGs were ultimately identified with significant diagnostic efficiency for TAAD, including CHEK2, HIF1A, HK2, HMGA1, SERPINA1, PTPN1, SLC2A1 and VEGFA. All these biomarkers were significantly upregulated in TAAD patients and their high-expressions were repetitively validated in multiple external datasets. Interestingly, there are some classical biomarkers reported to be involved in the acknowledged AD-associated mechanism. For example, by activating the HIF-1A pathway, hypoxia can promote the up-regulation of MMP-2/9 and further participate in the formation of AD and VEGFA also participates in the pathogenesis of AD via angiogenesis and remodeling [31]. HMGA1 has been found associated with the development of aortic aneurysm and dissection (AAD) via transcriptome-wide association study (TWAS) analysis [32] and SERPINA1 has been identified as a reliable molecular marker for the early diagnosis of AD patients [33]. HK2 is also considered as one of biomarkers to predict the degree of arterial 18F-fluorodeoxyglucose (FDG) accumulation in carotid disease in patients [34]. These studies further interpret the potential diagnostic capacity of diagnostic ARGs for TAAD.

Notably, we also screened vital targeted miRNAs to construct the complex regulatory networks for above 8 diagnostic ARGs based on the prediction of databases and validation in the dataset of non-code RNA-seq. The regulatory role of fatal miRNAs was extremely complicated with homodromous or reversed regulations. We found that five homodromous miRNAs (has-miR-124-3p, has-miR-128-3p, has-miR-155-5p, has-miR-195-5p and has-miR-23b-3p) were significantly upregulated while four reversed miRNAs (has-miR-1-3p, has-miR-145-5p, has-miR-20a-5p and has-miR-34a-5p) were downregulated in TAAD patients.

Immune cell infiltration has been raising the focus on the field of cardiovascular diseases in recent decades and macrophages have been found recalled and activated inside the aortic walls to be involved in the pathogenesis of TAAD through neoangiogenesis and matrix degradation [35]. In addition, through flow cytometry experiments, Flavia et al. identified the increase of NK cells and macrophages with the decrease of total T lymphocytes and T helper fractions in TAAD patients [36]. In this study, we drawn the immune cell landscape of TAAD by ssGSEA algorithm and demonstrated that innate immune-associated cells were more dominant than adaptive immune-related cells in tissues of TAAD, especially in macrophages, monocytes, activated dendritic cells and CD56+ dim NK cells. These results were consistent with previous studies and further verified in scRNA-seq datasets. In a recent scRNA-seq study, zhang et al. also identified specific cellular subtypes of macrophages and provided a preliminary evaluation of macrophages’ role in the development of TAAD [37]. Through integrated scRNA-seq analysis, we also investigated the functional enrichments of each cell in TAAD patients and identified the activation of multiple inflammatory-related signaling in B cells, fibroblasts, macrophages and monocytes including TNF, JAK-STAT and inflammation signaling. However, the inflammatory activation was not observed in T cells and significantly inhibited in SMC, suggesting the muscular damage and degradation of ECM.

Metabolic reprogramming refers to the adaptation of cellular metabolism in response to changes in environmental and physiological conditions, which is essential for maintaining cellular homeostasis in various pathological conditions, such as cancer, obesity, and diabetes [38]. Besides inflammatory-related pathways, immune cells, especially for macrophages and monocytes, exhibited significant activation of hypoxia and glycolysis process but with inhibition of oxidative phosphorylation, indicating the existence of metabolic reprogramming in macrophages and monocytes of TAAD patients. Lian et al. has also demonstrated that macrophages metabolic reprogramming could activated HIF-1α and ADAM17 signaling to aggravate AD via promoting vascular inflammation, elastic plate breakage and extracellular matrix degradation [39]. Moreover, we further compared the levels of anoikis scores among different cells and found the difference of anoikis scores between TAAD and HC cohorts was majorly dependent on macrophages and monocytes, suggesting the activation of anoikis in macrophages and monocytes might be fatal for TAAD. The differentiation trajectory analysis further identified the potential correlation among different macrophage subtypes and GSEA enrichment revealed the activation of inflammatory and metabolic reprogramming was dominated by macro1-3 and mono1/3/5 subtypes. Based on the expressional evolution of diagnostic ARGs on the trajectory, four vital ARGs, including HIF1A, HMGA1, SERPINA1 and VEGFA, were identified along with the changes of differentiation trajectory, and major expressions were conformably concentrated on macro1-3, mono1-2 and mono4 subtypes, implying these subtypes might participate in the activation of ARGs in TAAD patients.

However, there are still several limitations in our study. For one thing, although we have identified and validated eight diagnostic ARGs with high diagnostic efficiency, the number of each dataset is still limited and they were obtained from public databases. Hence, their corresponding diagnostic efficiency remains to be further explored through more external researches or clinical practice. For another, multiple immune cells, particularly in macrophages, were found significant association with anoikis scores in TAAD, indicating that macrophages subtypes might be responsible for the difference of anoikis. However, the concrete mechanism of macrophages leading to the development of TAAD by anoikis -related pathways remains to be further investigated and validated by more functional experiments *in vivo* and *in vitro*.

## CONCLUSIONS

Our study first screens diagnostic ARGs as the novel biomarkers for the precise diagnosis of TAAD based on integrated analysis. A total of eight diagnostic ARGs were successfully identified with high diagnostic efficiency and their functional annotations were applied to expound potential mechanism of anoikis in TAAD. Integrated RNA-seq and scRNA-seq analysis revealed that inherent immunity-related cells, especially for macrophages and monocytes, were considered as primary pathogenic parts for the development of TAAD, with close connection to the anoikis process. These findings provide a promising diagnostic biomarker for the accurately diagnosing the disease and would be helpful to further explore the potential pathogenesis with anoikis process for TAAD.

## Supplementary Material

Supplementary Table 1

Supplementary Table 2

Supplementary Table 3

Supplementary Table 4

Supplementary Table 5

Supplementary Table 6

Supplementary Table 7

Supplementary Table 8

Supplementary Table 9

Supplementary Table 10

Supplementary Table 11
