# Supplementary material for: Integrated bulk and scRNA sequence identified anoikis-related diagnostic biomarkers and potential association with immune infiltration in type A aortic dissection
Source: Aging (Albany NY). 2023 Oct 24;15(20):11268–85. doi: 10.18632/aging.205126 (PMC10637813; doi:10.18632/aging.205126)
Supplement: Supplementary Table 1 [file aging-15-205126-s001.pdf]

SUPPLEMENTARY TABLE

Supplementary Table 1. Datasets used in this study.

GEO id	Samples	Type	Role
GSE153434	10 TAAD and 10 HC	bulk RNA-seq	Training sets
GSE98770	6 TAAD and 5 HC	bulk RNA-seq	Test sets
GSE52093	7 TAAD and 5 HC	bulk RNA-seq	Test sets
GSE190635	4 TAAD and 4 HC	Whole-genome RNA-seq	Test sets
GSE213740	6 TAAD and 3 HC	single-cell RNA-seq	Exploring sets
